# A Multi-Omics Approach for Rapid Identification of Large Genomic Lesions at the *Wheat Dense Spike* (*wds*) Locus

**DOI:** 10.3389/fpls.2022.850302

**Published:** 2022-04-13

**Authors:** Zhenyu Wang, Shu Tao, Shaoshuai Liu, Meiling Jia, Dada Cui, Guoliang Sun, Zhongyin Deng, Fang Wang, Xingchen Kong, Mingxue Fu, Yuqing Che, Ruyi Liao, Tao Li, Shuaifeng Geng, Long Mao, Aili Li

**Affiliations:** ^1^National Key Facility for Crop Gene Resources and Genetic Improvement, Institute of Crop Science, Chinese Academy of Agricultural Sciences, Beijing, China; ^2^College of Agriculture, Yangzhou University, Yangzhou, China

**Keywords:** wheat, dense spike, RNA-seq, exome capture sequencing, *TaBUL1*

## Abstract

Optimal spike architecture provides a favorable structure for grain development and yield improvement. However, the number of genes cloned to underlie wheat spike architecture is extremely limited. Here, we obtained a wheat dense spike mutant (*wds*) induced by ^60^Co treatment of a common wheat landrace Huangfangzhu that exhibited significantly reduced spike and grain lengths. The shortened spike length was caused by longitudinal reduction in number and length of rachis cells. We adopted a multi-omics approach to identify the genomic locus underlying the *wds* mutant. We performed Exome Capture Sequencing (ECS) and identified two large deletion segments, named 6BL.1 at 334.8∼424.3 Mb and 6BL.2, 579.4∼717.8 Mb in the *wds* mutant. RNA-seq analysis confirmed that genes located in these regions lost their RNA expression. We then found that the 6BL.2 locus was overlapping with a known spike length QTL, qSL6B.2. Totally, 499 genes were located within the deleted region and two of them were found to be positively correlated with long spike accessions but not the ones with short spike. One of them, *TraesCS6B01G334600*, a well-matched homolog of the rice *OsBUL1* gene that works in the Brassinosteroids (BR) pathway, was identified to be involved in cell size and number regulation. Further transcriptome analysis of young spikes showed that hormone-related genes were enriched among differentially expressed genes, supporting *TraesCS6B01G334600* as a candidate gene. Our work provides a strategy to rapid locate genetic loci with large genomic lesions in wheat and useful resources for future wheat study.

## Introduction

Spike morphology is a complex multi-component trait determined by a series of related traits such as spike length, spikelet number, and spikelet density (Zhou et al., 2017). Spike length directly affects spikelet per spike and grains per spike, and hence final grain yield. Therefore, the identification of genes controlling spike-related traits is necessary to elucidate the genetic basis of wheat yield (Deng et al., 2017). Phytohormones are among the major factors that regulate spike morphogenesis (Barazesh and McSteen, 2008; McSteen, 2009). Brassinosteroids (BRs), gibberellins (GAs), and ethylene (ETH) are actively involved in panicle development and seed set, whereas auxins and strigolactones (SLs) inhibit axillary bud growth and impact effective panicle numbers (McSteen, 2009; Shimizu-Sato et al., 2009; Dun et al., 2012). Cytokinins (CKs), auxins, and jasmonic acid (JA) are also involved in meristem fate determination. An intricate network of hormonal pathways regulate panicle development and modulate spike/panicle architecture (Zhang and Yuan, 2014).

Transcription factors also play important roles in spike morphology (Bommert and Whipple, 2018; Wang et al., 2021). In rice, the *LARGE2-APO1/APO2* module controls panicle size and grain number and is a promising target for yield improvement (Huang et al., 2021). In barley, *HvMADS1* was found to be responsible for maintaining the unbranched spike architecture at relatively high temperatures (Li et al., 2021a), while the *AP2L-5* like proteins are evolutionarily conserved in grasses and able to promote inflorescence meristem activity and to restrict floret number per spikelet (Zhong et al., 2021). In wheat, the domestication gene *Q* participated in spike length and morphology (Faris et al., 2003; Sormacheva et al., 2015). The wheat *TEOSINTE BRANCHED1* (*TB1*) homolog also affects inflorescence architecture and development (Dixon et al., 2018). Recently, the AP2 transcription factor *WFZP* was reported to directly activate *VERNALIZATION1* (*VRN1*) and wheat *HOMEOBOX4* (*TaHOX4*) to regulate spikelet initiation and development (Li et al., 2021b). More importantly, *WFZP-D* was found to be a favorable gene for high-yield crop breeding (Du et al., 2021). In spite of this, research concerning wheat spike development is still quite limited and more genes should be exploited. More recently, Pang et al. (2020) conducted a large-scale genome-wide association study (GWAS) using a panel of 768 wheat cultivars, 327,609 single-nucleotide polymorphisms (SNPs) were generated by genotyping-by-sequencing and 395 quantitative trait loci (QTLs) were detected related to 12 traits in 7 environments, of which 26 QTLs were involved in spike length. These QTLs provided a basis for further discovery of spike architecture determining genes.

The availability of the high-quality reference genome of wheat allows the application of multiple genomics tools to expedite the identification of novel genes that affect important agronomic traits. Exome capture sequencing (ECS), which captures the coding regions of the genome, is suitable for the wheat genome due to its large size (He et al., 2019; Dong et al., 2020). A genomic locus was identified on chromosome arm 4BS using this method to be associated with plant height (Mo et al., 2018). Here, through identifying a dense spike mutant *wds* that was derived from ^60^Co treatment, we present a combinatorial approach that can locate candidate genes in a rapid way. Firstly, ECS identified two large deletion segments which were further confirmed by RNA-seq analysis. Then, based on known QTLs for wheat spike length, a candidate deletion region was isolated. In the end, combining correlation analysis of expression levels with long and short spike accessions, a gene whose expression level positively correlated with spike length was identified as a candidate for *wds*. The work provides an example of rapid identification of a large mutant locus and possible underlying genes.

## Materials and Methods

### Plant Materials

Common wheat landrace Huangfangzhu (HFZ or WT) and its ^60^Co induced wheat dense spike (*wds*) mutant (M5 lines) were kindly provided by Dr. Tao Li, Yangzhou University. Plants were grown at the field of Dongpu Experimental Station in Beijing (39.97°N, 116.34°E). Twenty wheat varieties from Chinese wheat mini-core collection, of which 10 varieties carried longer spikes and 10 varieties had shorter spikes. They were used for the association analysis of candidate gene expression levels relative to spike length traits (Wang et al., 2012). These wheat accessions were kindly provided by Dr. Xueyong Zhang, Institute of Crop Science, Chinese Academy of Agricultural Sciences, Beijing, China.

### Morphological and Cellular Analysis

Three spikes per plant of a minimum of 10 lines of WT and the mutant were selected for phenotyping. The middle section of spikes at W8.5 (Waddington stage 8.5) stage was collected for cytological observations. Sections of 8 μm were prepared longitudinally along the spike axis by using Leica Ultracut rotary microtome. The number of rachis cells was counted by selecting all cells from one node and cell lengths were measured by selecting the similar regions on the rachis of the mutant and WT spikes. The WSEEN Grain Test System (WSeen)^1^ was used to measure grain length, grain width, and thousand-grain weight.

### Exome Capture Sequencing

Genomic DNA was isolated from wheat leaves of HFZ and *wds* (M5 lines) using the CTAB method (Rogers and Bendich, 1985). The quality and quantity of the DNA was verified using 1.0% agarose gels and a NanoDrop 2000 spectrophotometer. ECS was performed using a standard protocol on libraries generated from 500 ng genomic DNA for all individuals. The libraries were constructed according to the manufacturer’s specifications (Illumina) and sequenced using the Illumina HiSeq X-ten platform to generate 150-bp paired-end reads. To avoid artificial bias, we removed following types of reads: (i) reads with ≥ 10% unidentified nucleotides (N); (ii) reads with > 10 nt aligned to the adaptor, with ≤ 10% mismatches allowed; (iii) reads with > 50% bases having phred quality < 5. High-quality reads. These reads were subsequently aligned to the genome of the IWGSC RefSeq v1.0 reference genome with the BWA software with the command “mem -t 10 -k 32 –M” (Li and Durbin, 2009). After alignment, improperly aligned unique paired-end reads (including secondary hits reads) were filtered out using samtools software with the command “samtools view -@ 10 -h -q 10 -f 2 -F 256” (Li et al., 2009). Consequently, SNP calling was performed using the Genome Analysis Toolkit (GATK, version v4.1) by the HaplotypeCaller method (McKenna et al., 2010).

### RNA-Seq Analysis

Total RNA was isolated from young spikes using TRIzol reagent (Invitrogen) at three key stages of HFZ and *wds*, W4, W6, and W8.5 of the Waddington scale with three biological replicates. For the long and short spike pools, ten accessions with the long spike in length distribution of a natural population (Guo et al., 2018a) were selected as members of “long spike pool” and 10 accessions with shorter spikes were selected as members of the “short spike pool.” Total RNA was isolated from young spikes at the W5.5 stage. Sequencing was performed on the Illumina HiSeq 2000 platform. An average of 15.5 Gb 150-bp pair-end clean reads were generated for each sample after filtering to remove low quality reads. Clean reads were aligned to the IWGSC RefSeq v1.0 reference genome using HISAT2 (v 2.1.0) with the command “hisat2 -p 8 –rna-strandness RF” (Kim et al., 2019). The unique and high quality mapped reads were retained for subsequent analysis by screening the flags “NH:i:1” and “quality value > 60” in aligned bam file. HTSeq was used to calculate read numbers mapped to the gene models (Anders et al., 2015). Read counts were then normalized into FPKM (Fragments Per Kilobase of transcript per Million mapped reads) to acquire relative expression levels using home-made Perl scripts. In the subsequent analysis, samples with very poor repeatability (*r*^2^ < 0.90) were removed in analysis.

Differential expression analysis was performed using limma R packages (Ritchie et al., 2015). In the process of limma analysis, RNA-seq reads with high quality were converted to the log-scale and empirically estimated for mean-variance relationship. The mean-variance trend was converted by the voom function into precision weights, which were incorporated into the analysis of log-transformed RNA-seq counts using the same linear modeling commands (Ritchie et al., 2015). Empirical Bayes moderated t-statistics and their associated *p*-values were generally used to evaluate the significance of the observed expression levels. After Benjamini-Hochberg’s adjustment, expressed genes with *p*-value < 0.05 were treated as differential expression genes.

### Statistical Analysis

Statistical analysis of mutant and HFZ lines was carried out *via* an independent Student’s *t*-test. The correlation coefficient between gene expression and spike length was calculated using the R function “cor()” based on Pearson’s method. The phenotypic data of grain length, grain width, and thousand-grain weight were obtained in multiple times/environments.

## Results

### Morphological Observation of the *wds* Mutant

**FIGURE 1:**
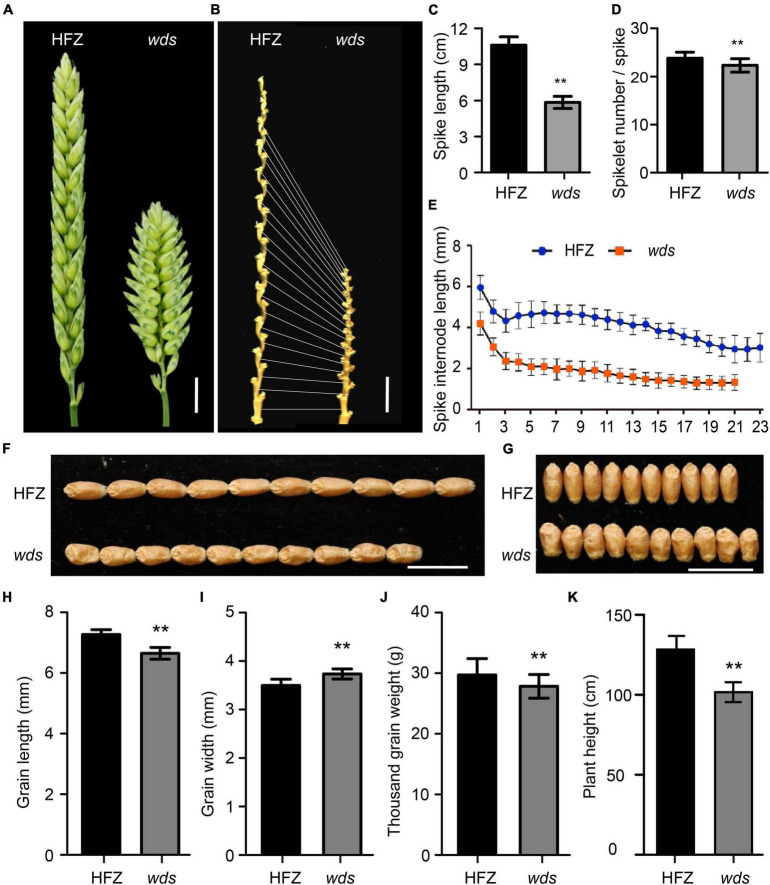
Morphology of the *wds* mutant. **(A)** The dense spike of the mutant. Scale bar = 1 cm. **(B)** Spike internode morphology. Scale bar = 1 cm. **(C,D)** Statistical analysis of spike length **(C)** and spikelet number per spike **(D)** of HFZ and *wds.* **(E)** Statistical analysis of spike internode length in HFZ and *wds*. **(F,G)** Comparison of grain length **(F)** and grain width **(G)** between HFZ and *wds*. **(H–K)** Statistical analysis of grain length **(H)**, grain width **(I)**, thousand grain weight **(J)**, and plant height **(K)** of HFZ and *wds*. n = 50. Significance was measured using Student’s *t*-test, **p < 0.01.

The *wds* mutant line at its M5 generation had significantly shorter spikes compared to the WT plants (41.4 vs. 95.5 mm, *p* < 0.01), only 56.7% of that of the WT (Figures 1A–C). The number of rachis internode representing spikelet number per spike was reduced from 23 in WT to 21 in the mutant (*p* < 0.01) (Figures 1B,D), with the average length of spike rachis internodes being reduced 47.5% from 4.15 to 1.97 mm (Figure 1E). On the other hand, grain length was reduced by 8.5% or 0.62 mm relative to WT (Figures 1F,H). However, the *wds* mutant exhibited increased grain width, while its thousand grain weight decreased relative to the WT (Figures 1G,I,J). The plant height of *wds* was also reduced by 20.8% (26.7 cm, *p* < 0.01) relative to WT (Figure 1K). Thus, the *wds* mutant showed systematic longitudinally shortened phenotypes, with shortened plant height, spike length, and grain length.

**FIGURE 2:**
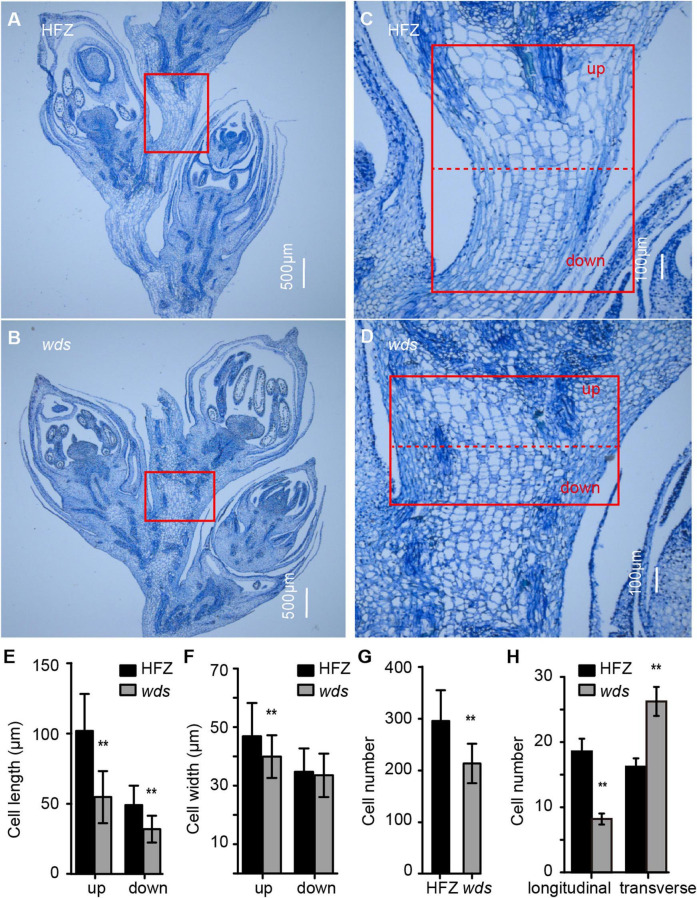
Cytological observations of rachis cells. **(A–D)** Magnified views of rachis cells in HFZ (up) and *wds* (down) in the longitudinal direction. **(A,B)** Scale bar = 500 μm. **(C,D)** Scale bar = 100 μm. **(E–H)** Statistical analysis of cell length, cell width, cell number and the number of cells in longitudinal and transverse directions. Significance was measured using Student’s *t*-test, **p < 0.01.

To gain insight into the causes of short spike length, we sectioned the rachis at W8.5 (Waddington staging) for microscopic observation and found that the length of mutant cells was reduced by 46.2% at the top and 34.9% at the bottom regions of the rachis (Figures 2a–d,e). In addition, cell width was observed to be reduced, but were mostly restricted to the upper part of the rachis (Figure 2f). Meanwhile, the number of rachis cells was significantly decreased, from 295.7 to 213.18 (*p* < 0.01) in *wds* (Figure 2g). Further comparison of longitudinal and transverse sections showed that the number of mutant rachis cells significantly decreased, from 18.6 ± 1.9 to 8.2 ± 0.8, in longitudinal sections, while increased from 16.3 ± 1.3 to 26.2 ± 2.2 in transverse sections (*p* < 0.01) (Figure 2h), suggesting that the re-organization of rachis cells in the internode may contribute to the altered mutant phenotypes. These data demonstrated that the shortened rachis was caused by the reduced length and number of rachis cells in the longitudinal direction.

### Identification of Two Major Deletions on Chromosome 6BL in *wds*

**FIGURE 3:**
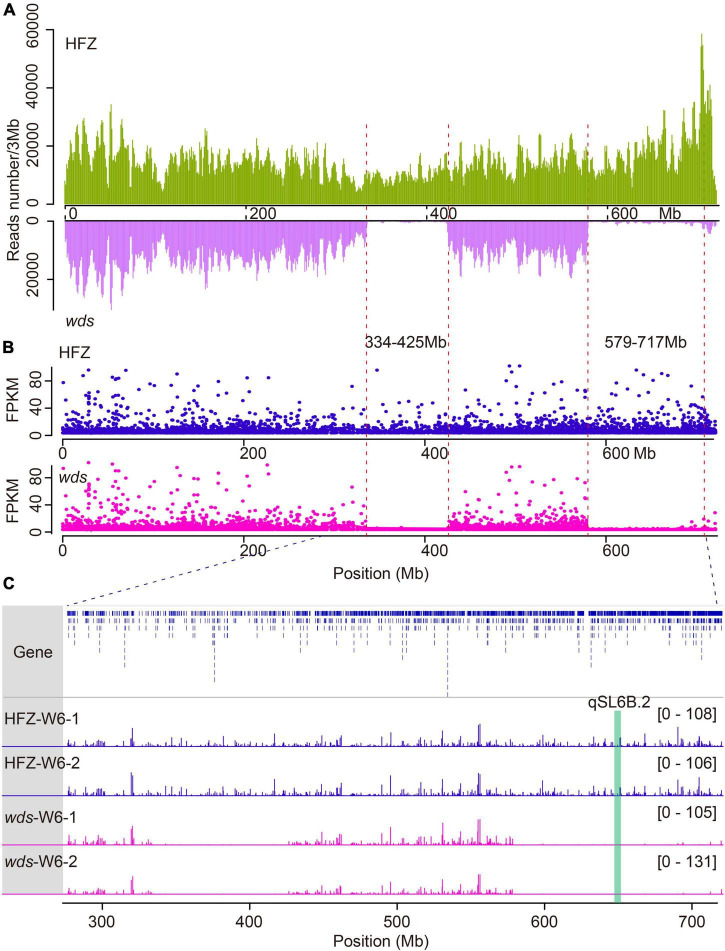
Comparison of genes expression and reads coverage within deleted regions in WT and *wds*. **(A)** Reads mapping depth of ECS reads on chromosome 6B. The horizontal axis (X-axis) corresponds to the length of the chromosome and the Y axis indicates reads mapping depth. **(B)** Gene expression levels in FPKM on Chr6B. **(C)** Reads coverage within deleted regions.

In order to probe the mutation loci in *wds*, ECS was performed using the first-generation wheat exome capture probes—which collectively represented 110 Mb of low copy number regions across the wheat genome (Jordan et al., 2015). Reads obtained were mapped to the IWGSC wheat genome assembly RefSeq v1.0 (Lang et al., 2018), yielding a total of 329.9 million mapping reads with an average of 98.9% mapping ratio for the two lines (Supplementary Table 1). By analyzing the dataset using the Genome Analysis Toolkit (GATK) pipeline, we identified a total of 2,024,173 SNPs and 185,396 short insertions and deletions (INDELs) (Supplementary Table 2). Surprisingly, two large segment deletions were observed on the long arm of chromosome 6B and were named 6BL.1 (334.8∼424.3 Mb) carrying 113 genes and 6BL.2 (579.4∼717.8 Mb) carrying 499 genes (Figure 3A and Supplementary Table 3).

To check whether these genes were lost as a result of segment deletion in *wds*, we further performed transcriptome analysis of the mutant line and the wild type. To cover more genes, we isolated RNA from spikes at three stages W4, W6, and W8.5 which represented the time points at the appearance of stamen primordium stage, the stylar canal of ovules with a narrow opening stage, and the formation of stigmatic branches stage, respectively (Zheng et al., 2016). These stages also corresponded to the times of pre-, middle-, and post-spike elongation (Figures 5A–C). An average of 15.5 Gigabyte (Gb) 150-bp pair-end clean reads were obtained for each sample using Illumina HiSeq 2000 platform (Supplementary Table 4). After data filtering, clean reads were mapped to the IWGSC wheat genome reference annotation (v1.0), yielding an average of 97.49% overall alignment rate and 90.08% of them were uniquely mapped to the wheat genome (Supplementary Table 5). Expression levels were obtained by mapping reads to the gene model and were converted to FPMK. Gene expression correlation analysis showed high coefficients among replications, more than 0.98 (Supplementary Table 6). Principal component analysis (PCA) confirmed the quality of the replications (Supplementary Figure 1). In line with the ECS result, there was only 0.45 and 0.9% sequencing coverage within the 6BL.1 and 6BL.2 regions in *wds*, which may be caused by mismatching, while the average coverage was 13.2 and 15.1% in the corresponding genomic regions in WT, confirming that these genes were indeed lost in *wds* (Figures 3B,C).

### Identification of a Candidate Gene in the Interval of 6BL.2

To check whether the two deleted segments were responsible for the mutant phenotype, we mapped two deleted segments with existing spike length QTLs on chromosome 6B and found that the 6BL.2 locus overlapped with QTL for spike length located at 643.8–644.2 Mb (*p* = 9.25E-06) as reported by a previous study (Pang et al., 2020), suggesting that a possible candidate gene may be located in this region.

**FIGURE 4:**
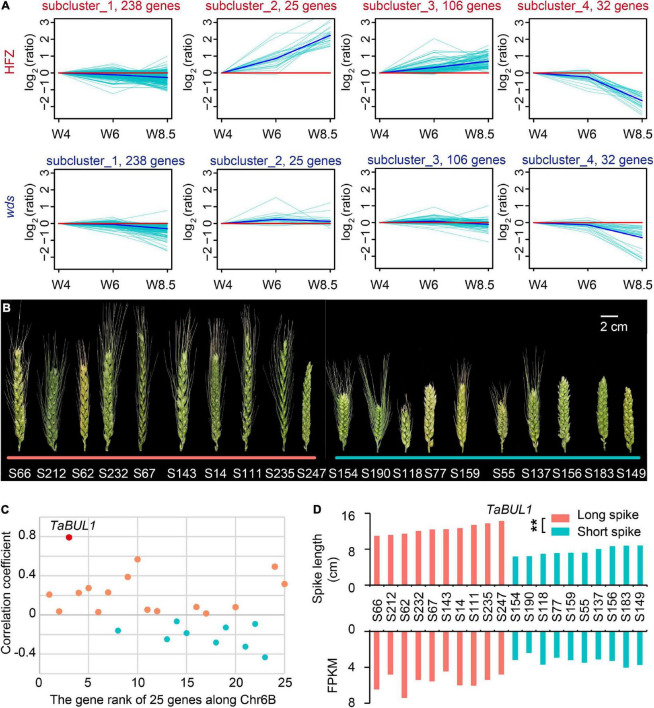
Characterization of candidate loci based on spike length pools. **(A)** Gene expression patterns on chromosome 6B in HFZ and *wds.* **(B)** Selected varieties for spike length pools. The left 10 accessions from the long spike pool and the right 10 from the short spike pool. Scale bar = 2 cm. **(C)** The correlation coefficient of the expression levels of 25 expressed genes with spike length using spike length pools. **(D)** Significant correlation between spike lengths and expression levels of *TaBUL1* in varieties of the two spike length pools. *p*-value was determined by Student’s *t*-test. **p < 0.01.

We then screened the 499 genes at 6BL.2 by studying expressed genes (401) with FPKM > 1 in at least one sample (Supplementary Table 7). *K*-means clustering divided these genes into four subclusters according to their expression patterns over the developmental course (Figure 4A). We paid special attention to subcluster 2 (containing 25 genes) because gene expression levels in this cluster were continuously increased that was consistent with spike development in WT, but not in *wds* (Figure 4A).

We then examined the expression patterns of these 25 genes for their association with spike length by using spike length pools composed of long- and short-spike accessions respectively. As shown in Figure 4, 10 accessions with the long spike in length distribution of a natural population (Guo et al., 2018a) were selected as members of “long spike pool” and 10 accessions with short spike in length distribution were selected as members of “short spike pool” (Figure 4B). Total RNA was isolated from spikes of these accessions at stage W5.5 and was used for sequencing and transcriptome analysis. Among the above 25 genes, there were only two genes whose expression level was not only significantly positively correlated with phenotype (*p* < 0.01), but also had correlation coefficient greater than 0.5 (Figure 4C and Supplementary Table 8). One of the two genes, *TraesCS6B01G334600*, was found to have the highest correlation between its RNA expression level and spike length with a 0.79 correlation coefficient (*p* < 0.01) (Figure 4C). Annotation showed that the gene, named *TaBUL1-6B*, was orthologous gene of the rice *OsBUL1* (*BRASSINOSTEROID UPREGULATED 1-LIKE1*) gene (Supplementary Figure 3). In addition to the orthologs of *OsBUL1* on homoeologous group (HG) 6 (*TraesCS6A01G306200*, *TaBUL1-6A*; *TraesCS6B01G334600*, *TaBUL1-6B*; *TraesCS6D01G285300*, *TaBUL1-6D*), *TaBUL1* indeed has paralog genes on HG 7 (*TraesCS7A01G185300*, *TaBUL1-7A*; *TraesCS7B01G090500*, *TaBUL1-7B*; *TraesCS7D01G187000*, *TaBUL1-7D*) (Supplementary Figure 3 and Supplementary Table 9). The expression patterns of *TaBUL1* on HG 6 (*TaBUL1-6A, TaBUL1-6B, TaBUL1-6D*) were obviously different from the ones on HG7 (*TaBUL1-7A, TaBUL1-7B, TaBUL1-7D*). From W4 to W6, *TaBUL1* on HG7 (7A, 7B, 7D) were nearly not expressed, while the ones on chromosome 6 were expressed up to about 10 FPKM, indicating the obvious subfunctionalization of these two group genes (Supplementary Figure 4A).

As for the three homoeologus genes *TaBUL1* (6A, 6B, 6D), *TaBUL1-6B* has one different amino acid in the conserved domain bHLH from the other two (Supplementary Figure 4B), suggesting the function of *TaBUL1-6B* on deletion region might be different from *TaBUL1-6A* and *TaBUL1-6D*. In addition, the *wds* mutant showed smaller spikelet and produced smaller grains relative to WT, similar to phenotypes of the *OsBUL1* mutant. More importantly, the expression level of *TaBUL1-6B* in long spike extreme pool was significantly higher (*p* < 0.0001) than that in short spike extreme pool materials (Figure 4D), while expression of *TaBUL1-6B* was barely detectable in the *wds* mutant. Thus, we deduced that *TaBUL1-6B* may be a candidate gene responsible for the altered phenotype of the *wds* mutant.

### Transcriptome Alteration Caused by the *wds* Mutation

**FIGURE 5:**
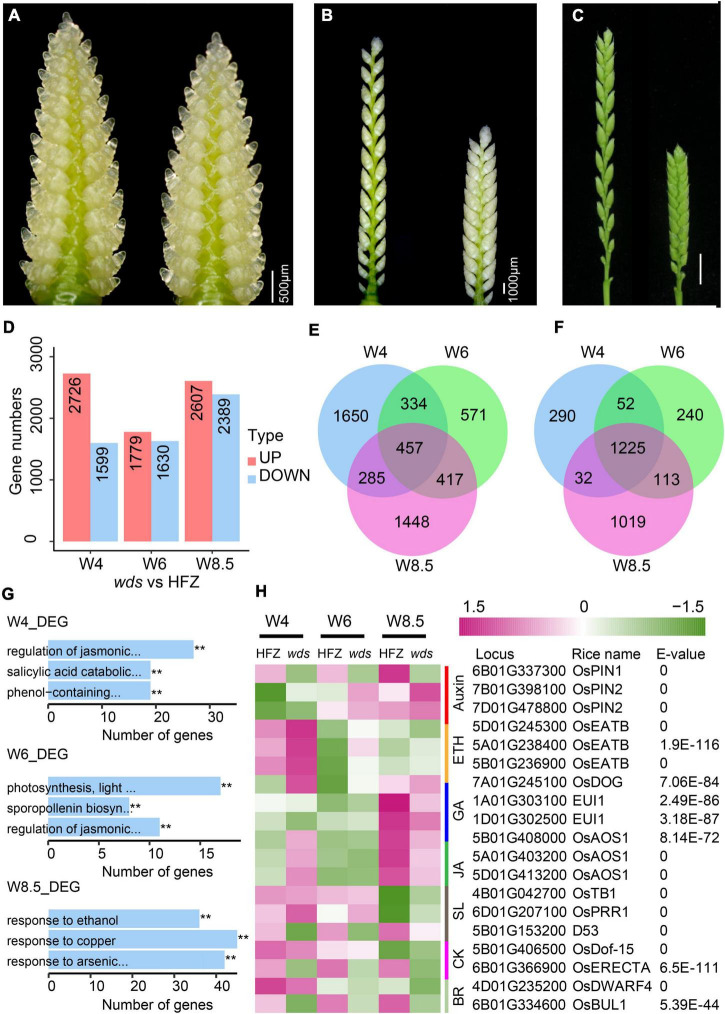
Differentially expressed genes (DEGs) between HFZ and *wds* spikes. **(A–C)** Stages of spikes used for RNA-seq analysis. **(A)** W4, **(B)** W6, and **(C)** W8.5. Scale bar = 500 μm **(A)**, Scale bar = 1000 μm **(B)**, Scale bar = 1 cm **(C)**. **(D)** Numbers of DEGs at stages W4, W6, W8.5. Red color, up-regulated; green color, down-regulated. Venn diagrams of up-regulated **(E)** and down-regulated **(F)** DEGs at W4, W6, and W8.5. **(G)** The top three most significantly enriched GO terms at the three developmental stages. **(H)** Heatmap of expression dynamics of known hormone-related genes. On the right are RefseqV1.0 gene annotation and similarity (blast *E*-values) of the genes with rice homologs.

To study genome-wide gene expression changes in the *wds* mutant, we analyzed RNA-seq data from spikes at W4, W6, and W8.5 of the WT and the mutant (Figures 5A–C). Cluster pedigree analysis showed highly correlated gene expression levels among replications (Supplementary Figure 2). A total of 2,726 (W4), 1,779 (W6), and 2,607 (W8.5) genes were significantly up-regulated (*p*-value < 0.05) and 1,599 (W4), 1,630 (W6), and 2,389 (W8.5) genes were significantly down-regulated (*p*-value < 0.05) in the indicated stages in the *wds* mutant (Figure 5D and Supplementary Tables 10, 11). Of these, 457 up-regulated genes and 1,225 down-regulated genes were shared at three developmental stages, whereas others were stage-specific (Figures 5E,F).

Gene Ontology (GO) analysis revealed three most enriched GO terms at W4, i.e., the JA-mediated signaling pathway (GO: 2000022, *p* = 4.04e-17), the SA-mediated signaling pathway (GO: 0046244: *p* = 6.68e-13), and the phenol-contained compound catabolic pathway (GO: 0019336, *p* = 1.35e-11) (Figure 5G and Supplementary Table 12). JA-mediated signaling pathway (GO: 2000022) was also enriched at W6 (*p* = 1.6e-13) where the other two most enriched GO terms were photosynthesis pathway (GO: 0009765, *p* = 2.54e-4) and sporopollenin biosynthetic pathway (GO: 0080110, *p* = 2.54e-4) (Figure 5G and Supplementary Table 13). Interestingly, at W8.5, genes mostly enriched were those related to ethanol (GO: 0045471, *p* = 1.03e-25), response to copper ion (GO: 0046688, *p* = 1.12e-20), and response to arsenic-containing substance (GO: 0046685, *p* = 1.20e-11) (Figure 5G and Supplementary Table 14), indicating that genes for spike development were mainly functional at early stages.

Further examination of known hormone-related genes affected by the *wds* mutation showed that most hormonal genes, such as GA, ETH, JA, SL, and auxin related genes, except those related to SA, were significantly altered in the mutant (Figure 5H). Of note, *TraesCS5B01G153200*, a homolog of *D53*, a governing gene of spike length in the regulation of spike architecture, was continuously down-regulated over the three stages. Besides, all three homoeologs of the rice *OsEATB* gene (*TraesCS5A01G238400*, *TraesCS5B01G236900*, *TraesCS5D01G24530*), which encodes an ERF protein and is involved in the crosstalk between ETH and GA to reduce rice plant height and panicle length at the maturity stage, were up-regulated at the stage of W4 in *wds*. Taken together, the *wds* mutant seemed to affect the complex hormone regulatory network through the BR pathway, probably *via TaBUL1*, making it the most possible candidate gene for the dense spike phenotype.

## Discussion

### A Multi-Omics Approach to Rapidly Locate a Candidate Causal Locus in a Mutant

Improving spike morphology is an effective strategy to increase grain yield (Guo et al., 2018b). This can be achieved by modifying spike length, spikelet number per spike, and the number of florets per spikelet. The discovery of functional genes underlying agronomic traits is of great importance for wheat improvement. Recently, an effective method, termed bulked segregant exome capture sequencing (BSE-Seq) was reported for identifying causal mutations or candidate genes which integrated the newly designed wheat exome capture panel, bulked segregant pools sequencing, and a robust algorithm varBScore (Dong et al., 2020). BSE-Seq was used to identify a wheat yellow leaf mutant gene, *ygl1*, using an ethyl methanesulfonate (EMS) mutant population which was found to encode a magnesium- chelatase subunit chlI (Dong et al., 2020). In this work, we combined ECS, RNA-seq, and association analysis with extreme trait pools to rapidly characterize a spike mutant *wds* that showed dense and short spikes. ECS identified two large deletion segments which were confirmed by RNA-seq. Assisted with gene expression data, we found the second deletion segment overlapped with a reported spike length QTL and identified a possible candidate gene based on its expression pattern and information from the model plant. We showed that in the case of large deletion, RNA-seq helps to rapidly detect potential loci and genes based on their expression levels. It may serve as the first step for gene cloning with unknown genetic lesions before investing unnecessary effort.

### Phytohormones and Spike Morphology Development

Phytohormones are small regulatory molecules that form a regulatory network in coordinating various developmental aspects of yield-related traits and therefore control the yield potential of crops (Zhang and Yuan, 2014). Phytohormones are also extensively involved in shoot branching (tillering), panicle branching, panicle length, and seed set percentage (Santner et al., 2009). JA plays a key role in spikelet morphogenesis, deciding floral organ identity and floral organ number along with the E-class gene, *OsMADS1* for floral meristem determinacy in spikelet development (Cai et al., 2014). Our transcriptome analysis showed the GO term for JA-mediated signaling pathway genes were enriched at both W4 and W6 stages, suggesting that JA signaling pathway was affected in the *wds* mutant. On the other hand, ETH also plays a role in the regulation of panicle architecture, controlling grain size and grain filling rate (Yin et al., 2017). At W8.5, in addition to three significantly enriched GO terms mentioned above, one GO term, response to ETH stimulus, is highly enriched (GO:0009727, *p* = 2.61e-6) (Supplementary Table 11). This shift from the JA pathway to the ETH pathway may imply that the *wds* mutant has a phasing effect on spike development. Most importantly, the candidate gene identified from the locus 6BL.2 was a BR-related gene, providing additional evidence that dense spike phenotype is related to hormones. In rice, *SMALL GRAIN 11* (*SMG11*), a novel allele of *DWARF2* (*D2*) encoding a cytochrome P450 (CYP90D2), is involved in BR biosynthesis. The morphological traits of *smg11*, including erect, shorter, and denser panicles at the mature stage, exhibit decreased length of rachis and more but smaller grains, suggesting that BR plays a role in deciding cell division and elongation. Mutation of BR-related genes disrupts regular cell division and elongation, resulting in a short spike phenotype (Fang et al., 2016). In our study, microscopic observations showed significantly decreased length of rachis cells and the increased number of transverse rachis cells in the *wds* mutant, consistent with the function of BR in regulating plant cell elongation and division. These data supported the hypothesis that a gene associated with BR was responsible for the phenotypic alteration. Further experiments should be carried out *via* gene editing strategy to confirm its function.

### The Candidate Gene May Have Pleiotropic Effects

In addition to spike length, the *wds* mutant also showed a clear reduction in plant height, indicating its nature as a pleiotropic gene. In rice, the *Ghd7* gene (*Grain number*, *plant height*, *and heading date7*), for example, encodes a CCT (CONSTANS, CONSTANS-LIKE, and TIMING OF CHLOROPHYLL A/B BINDING1) domain protein which is involved in the rice flowering pathway but also contributes to rice yield potential (Xue et al., 2008). In rice, *OsBUL1* was involved in the regulation of cell size development and the mutation of *OsBUL1* reduced plant height by making internode cells shorter (Jang et al., 2017). Similarly, *OsBUL1* was also involved in controlling plant height, spike length, and grain length (Heang and Sassa, 2012). We showed here that the wheat *TaBUL1* may also serve as a pleiotropic gene and be responsible for multiple traits in wheat. *TaBUL1-6B* was located in one of the two large deletion segments that overlapped with a known spike length QTL. RNA-seq analysis supported its function as a major regulator for spike development in wheat. The gene may be valuable in breeding for yield improvement.

## Conclusion

With a high-quality wheat genome sequence available, a number of techniques that were once only available for model plants can be applied to the genetic cloning agriculturally important genes of this polyploid species. This work not only provides a strategy for rapidly locating large lesions in wheat using multiple genomic methodologies, but also provides valuable resources for wheat research, including ECS data, transcriptome data and extreme pool data of critical stages of spike development.

## Data Availability Statement

The ECS and RNA-seq data have been submitted to NCBI under the project numbers PRJNA792309 and PRJNA803598.

## Author Contributions

AL, LM, and SG designed the project. MJ, ST, SL, DC, and GS performed laboratory experiments. TL, ZD, FW, XK, and SG aided in experiments. ZW, MF, YC, and RL performed data analysis. ZW and SL drafted the manuscript. AL and LM revised the manuscript. All authors have read and approved the manuscript.

## Conflict of Interest

The authors declare that the research was conducted in the absence of any commercial or financial relationships that could be construed as a potential conflict of interest.

## Publisher’s Note

All claims expressed in this article are solely those of the authors and do not necessarily represent those of their affiliated organizations, or those of the publisher, the editors and the reviewers. Any product that may be evaluated in this article, or claim that may be made by its manufacturer, is not guaranteed or endorsed by the publisher.

## Footnotes

1
http://www.wseen.com/

## Supplementary Material

Supplementary Figure 1 Principal component analysis (PCA) of RNA-seq samples, showing good replication quality.

Supplementary Figure 2 Cluster dendrogram analysis of RNA-seq samples.

Supplementary Figure 3 The phylogeny of wheat *BUL* genes.

Supplementary Figure 4 Expression and protein sequence characterization of *TaBUL1* gene. **(A)** The expression of *TaBUL1* in HFZ and *wds*. **(B)** The multiple sequence alignment among TaBUL1 protein sequences.
