# Supplementary figures and images for: A Multi-Omics Approach for Rapid Identification of Large Genomic Lesions at the Wheat Dense Spike (wds) Locus
Source: Front Plant Sci. 2022 Apr 13;13:850302. doi: 10.3389/fpls.2022.850302 (PMC9043957; doi:10.3389/fpls.2022.850302)

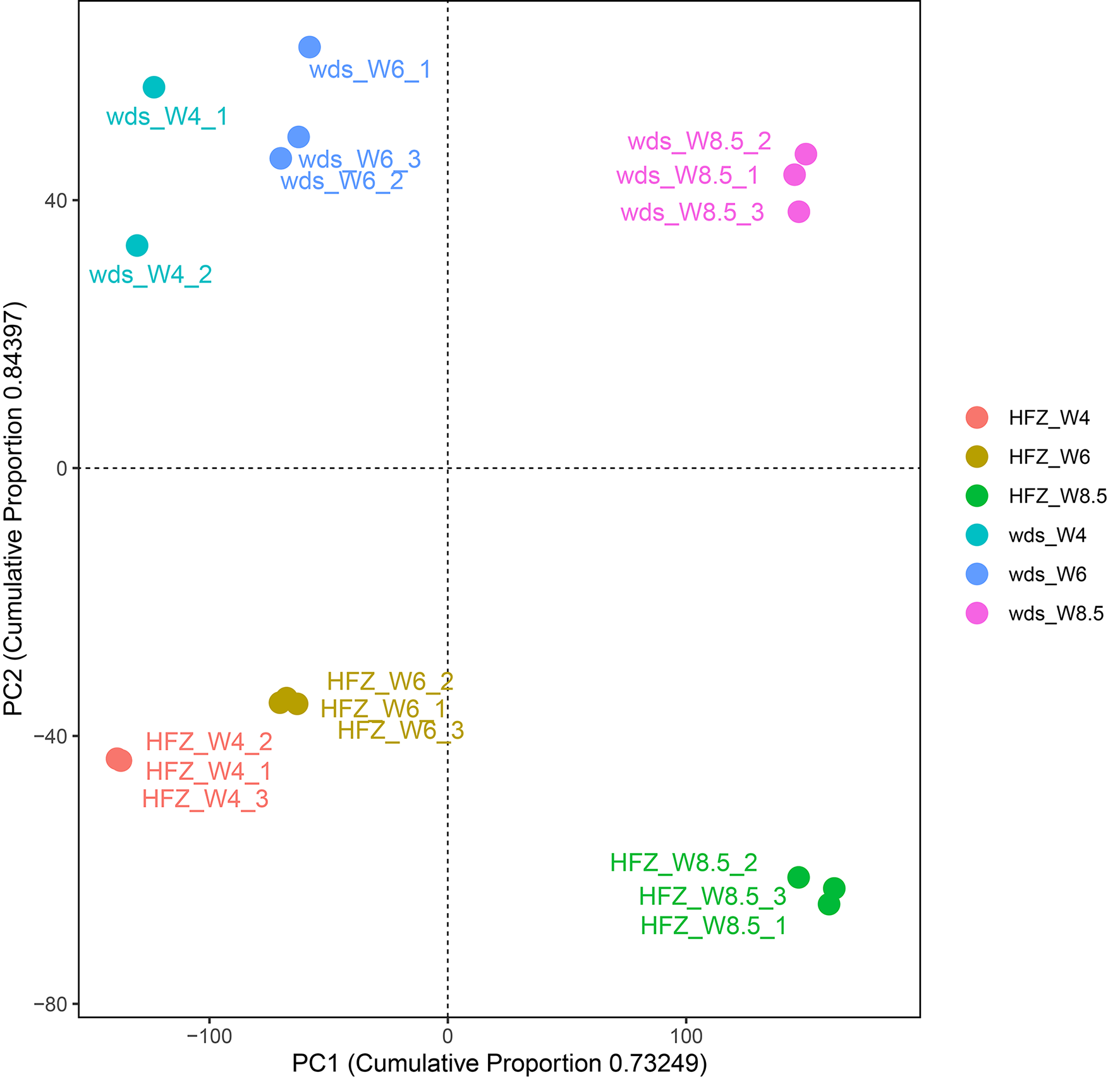

Supplement: Supplementary Figure 1 — Principal component analysis (PCA) of RNA-seq samples, showing good replication quality. [file Image_1.TIF]

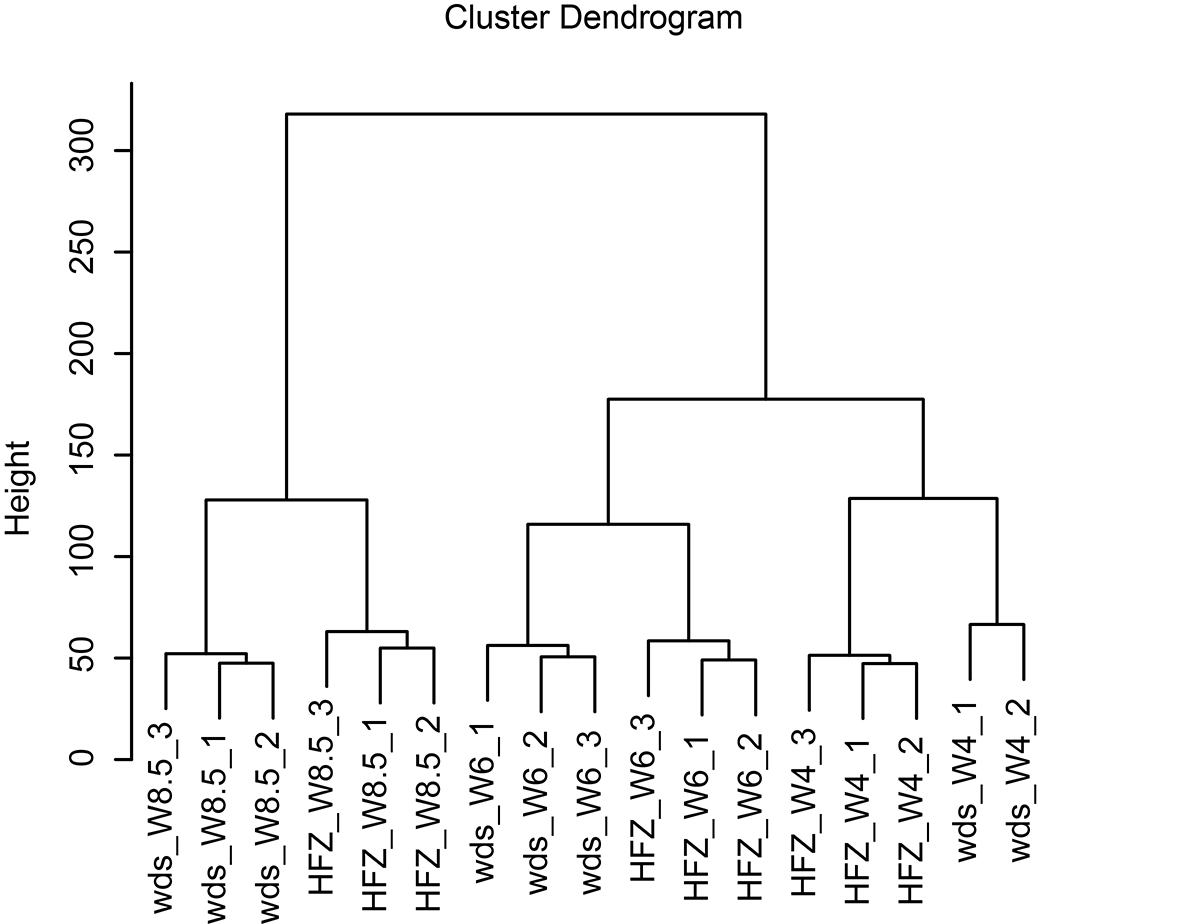

Supplement: Supplementary Figure 2 — Cluster dendrogram analysis of RNA-seq samples. [file Image_2.TIF]

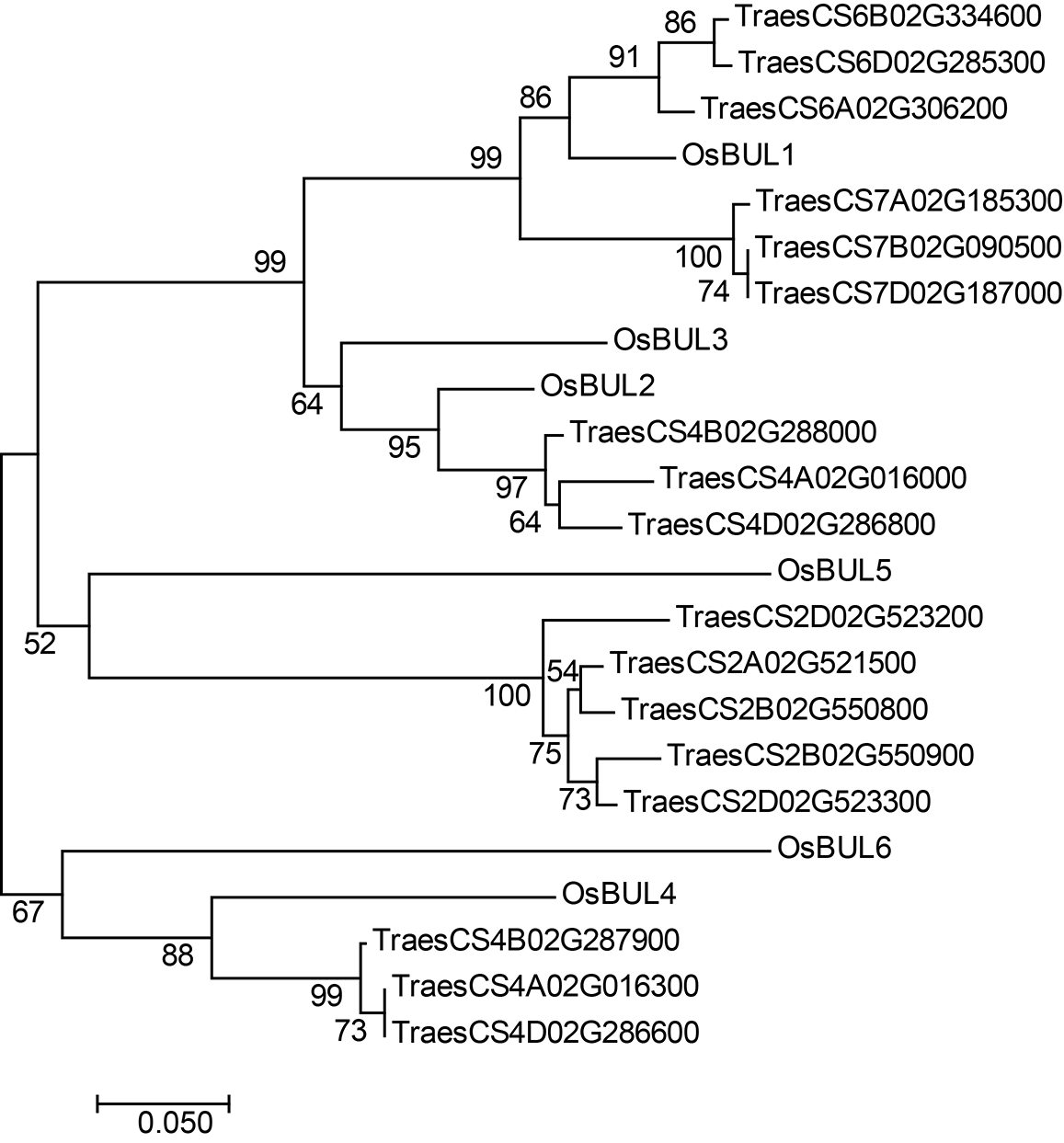

Supplement: Supplementary Figure 3 — The phylogeny of wheat BUL genes. [file Image_3.TIF]

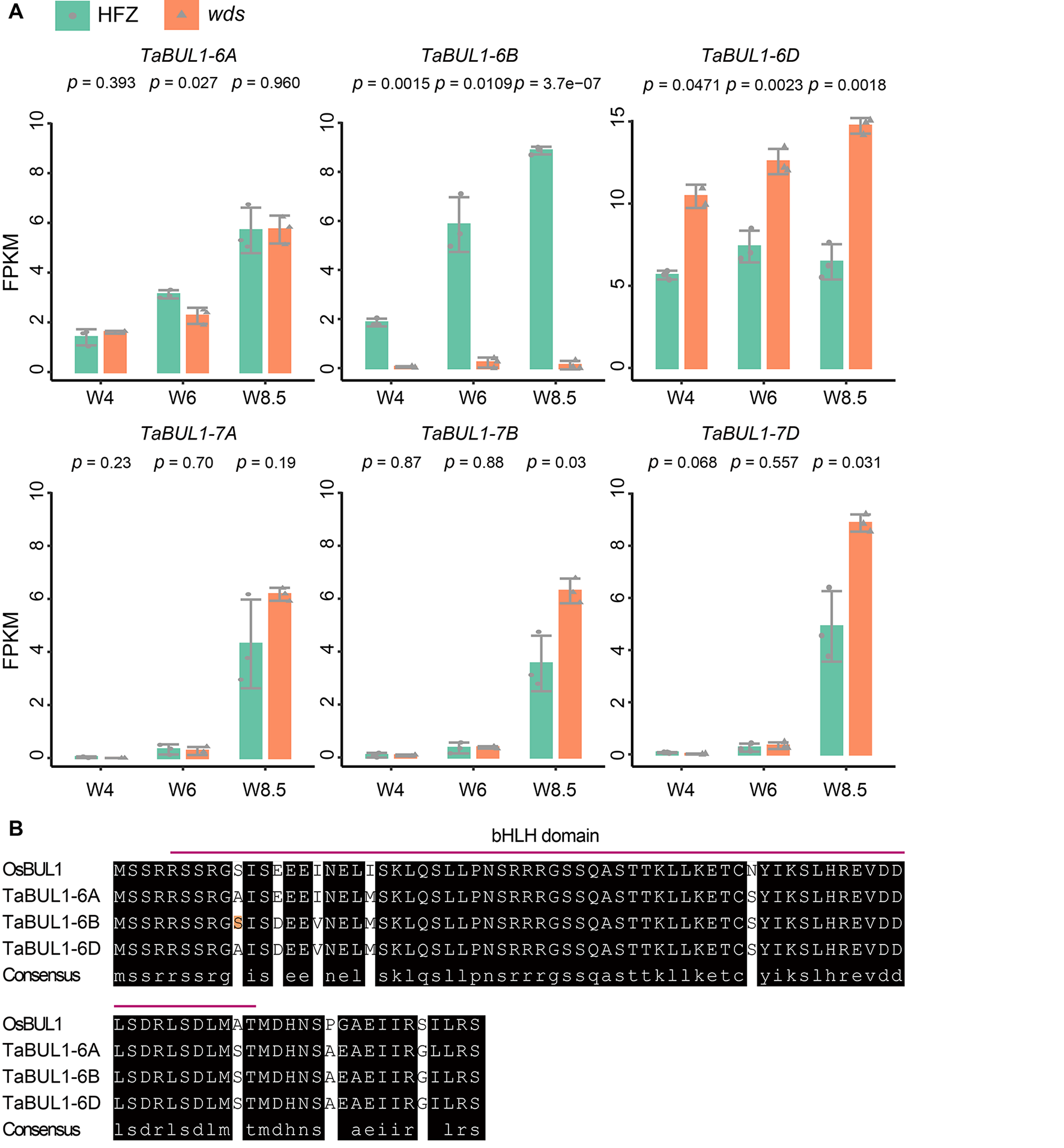

Supplement: Supplementary Figure 4 — Expression and protein sequence characterization of TaBUL1 gene. (A) The expression of TaBUL1 in HFZ and wds. (B) The multiple sequence alignment among TaBUL1 protein sequences. [file Image_4.TIF]
